# Identification of a Functional Small Noncoding RNA of African Swine Fever Virus

**DOI:** 10.1128/JVI.01515-20

**Published:** 2020-10-14

**Authors:** Laura E. M. Dunn, Alasdair Ivens, Christopher L. Netherton, David A. G. Chapman, Philippa M. Beard

**Affiliations:** a The Pirbright Institute, Pirbright, Surrey, United Kingdom; b The Roslin Institute and Royal (Dick) School of Veterinary Studies, University of Edinburgh, Roslin, Midlothian, United Kingdom; c Centre for Immunity, Infection and Evolution, University of Edinburgh, Edinburgh, United Kingdom; University of Illinois at Urbana Champaign

**Keywords:** African swine fever virus, porcine, small RNA

## Abstract

*African swine fever virus* (ASFV) causes a lethal hemorrhagic disease of domestic pigs, against which no vaccine is available. ASFV has a large, double-stranded DNA genome that encodes over 150 proteins. Replication takes place predominantly in the cytoplasm of the cell and involves complex interactions with host cellular components, including small noncoding RNAs (sncRNAs). A number of DNA viruses are known to manipulate sncRNA either by encoding their own or disrupting host sncRNA. To investigate the interplay between ASFV and sncRNAs, a study of host and viral small RNAs extracted from ASFV-infected primary porcine macrophages (PAMs) was undertaken. We discovered that ASFV infection had only a modest effect on host miRNAs, with only 6 miRNAs differentially expressed during infection. The data also revealed 3 potential novel small RNAs encoded by ASFV, ASFVsRNA1-3. Further investigation of ASFVsRNA2 detected it in lymphoid tissue from pigs with ASF. Overexpression of ASFVsRNA2 led to an up to 1-log reduction in ASFV growth, indicating that ASFV utilizes a virus-encoded small RNA to disrupt its own replication.

**IMPORTANCE** African swine fever (ASF) poses a major threat to pig populations and food security worldwide. The disease is endemic to Africa and Eastern Europe and is rapidly emerging into Asia, where it has led to the deaths of millions of pigs in the last 12 months. The development of safe and effective vaccines to protect pigs against ASF has been hindered by lack of understanding of the complex interactions between ASFV and the host cell. We focused our work on characterizing the interactions between ASFV and sncRNAs. Although comparatively modest changes to host sncRNA abundances were observed upon ASFV infection, we discovered and characterized a novel functional ASFV-encoded sncRNA. The results from this study add important insights into ASFV host-pathogen interactions. This knowledge may be exploited to develop more effective ASFV vaccines that take advantage of the sncRNA system.

## Abstract (precis)

African swine fever (ASF) poses a major threat to pig populations and food security worldwide. The disease is endemic to Africa and Eastern Europe and is rapidly emerging into Asia, where it has led to the deaths of millions of pigs in the last 12 months. The development of safe and effective vaccines to protect pigs against ASF has been hindered by lack of understanding of the complex interactions between ASFV and the host cell. We focused our work on characterizing the interactions between ASFV and sncRNAs. Although comparatively modest changes to host sncRNA abundances were observed upon ASFV infection, we discovered and characterized a novel functional ASFV-encoded sncRNA. The results from this study add important insights into ASFV host-pathogen interactions. This knowledge may be exploited to develop more effective ASFV vaccines that take advantage of the sncRNA system.

## INTRODUCTION

African swine fever (ASF) is a highly pathogenic viral disease of swine. Virulent strains cause acute hemorrhagic fever in domestic pigs, with mortality rates of up to 100% (1). There is currently no effective vaccine or treatment (2). ASF is caused by African swine fever virus (ASFV), the only member of the *Asfarviridae* family. The virus sits within the nucleocytoplasmic large DNA virus (NCLDV) superfamily, which also includes the *Poxviridae*. NCLDVs have large, double-stranded DNA genomes and replicate predominantly in the cytoplasm of infected cells (3). ASFV replicates in cells of the monocyte/macrophage lineage. This predominantly occurs in the cytoplasm of infected cells, although an early stage of DNA replication has been identified in the nucleus (4). ASFV evades the antiviral defenses in these cells by modulation of a number of host cell pathways, including type I IFN induction (5), apoptosis (6), host-cell protein synthesis (7), and the NF-κB and NFAT signaling pathways (8). Descriptions of these host-cell interactions are reviewed in detail in reference 9. The nuclear phase of replication has been shown to activate the host nuclear DNA damage response (DDR) (10) and also leads to alteration of nuclear architecture by the disruption of subnuclear domains and chromatin texture (11).

Small noncoding RNAs (sncRNAs) are classes of small RNA (<200 nucleotides [nt]) that are involved in the regulation of gene expression and genome stability, predominantly through RNA interference (RNAi) mechanisms. Eukaryotic cells produce multiple classes of sncRNA, including microRNAs (miRNAs), PIWI-interacting RNAs (piRNAs), and endogenous small interfering RNAs (siRNAs) (12). These sncRNAs are involved in many biological processes, including apoptosis, differentiation, stress response, and immune activation (13). Therefore, it is unsurprising that viruses manipulate and exploit sncRNAs for their own benefit. Virus-encoded miRNAs have been identified in a number of DNA virus families, including *Herpesviridae*, *Polyomaviridae*, *Iridoviridae*, *Ascoviridae*, *Baculoviridae*, and the *Adenoviridae* (14). These miRNAs play a variety of roles, including cell proliferation regulation, control of apoptosis, and modulation of host immunity (15). For example, the Kaposi’s sarcoma-associated herpesvirus (KSHV)-encoded miRNA, miR-K1, regulates the switch between lytic and latent viral replication by control of NF-κB expression via targeting of the host IκBα transcript (16). Other classes of viral ncRNAs have also been identified (reviewed in reference 17). An interesting example is the herpes simplex virus 1 (HSV-1)-encoded non-miRNA small RNAs (LAT sRNA1 and sRNA1) that regulate productive infection and inhibit apoptosis (18).

DNA viruses have also been shown to manipulate host sncRNAs by targeting specific host miRNAs, as in the case of murine cytomegalovirus (MCMV) infection. MCMV induces degradation of cellular miR-27a and miR-27b, which are important for MCMV replication *in vivo* (19). A more nonspecific and global effect is wrought by vaccinia virus (VACV), the prototypic poxvirus and NCLDV member, which induces widespread disruption of host miRNAs by a process of 3′ polyadenylation and decay (20, 21). As RNAi is the major antiviral pathway in invertebrates, a number of arthropod-borne (arboviruses) are known to manipulate sncRNA during replication to evade this immune response. Interestingly, ASFV is currently the only known DNA arbovirus, and it replicates in the soft tick vector of the *Ornithodoros* spp., which have a functional RNAi system (22). Overall, it is apparent that manipulation of sncRNA systems is a common feature of viruses to further their survival, replication, and pathogenesis.

In addition, the engineering of miRNA targeting has been developed as a novel method to attenuate viral replication. This method involves the engineering of a virus with host miRNA target sites in viral RNA, which is then subjected to miRNA silencing (23). Originally, this methodology was limited to the attenuation of RNA viruses, including poliovirus (24), Dengue virus (25), and human norovirus (26), with the miRNA target sites added directly to the viral RNA genome. However, it has also been shown to be an effective method of attenuating DNA virus replication, including VACV (27) and human cytomegalovirus (27), by the targeting of specific transcripts. There is no commercially available vaccine against ASFV, and current live-attenuated vaccine (LAV) development relies on the deletion of viral genes associated with virulence and/or immune evasion. There has been some success with these methods (28, 29), but as only nonessential genes can be deleted, a challenge remains in selecting genes to get the correct balance between attenuation and virulence. Therefore, miRNA targeting could provide a novel method of attenuation that can be used in parallel with gene deletion. However, the use of host miRNAs to target the virus would require that host miRNAs are not disrupted during infection. Therefore, we sought to investigate the interaction between ASFV and sncRNAs with the aim of gaining important insights into host-pathogen interactions, knowledge of which could be used to optimize LAV ASFV vaccines.

The effect of ASFV infection on host miRNAs has been investigated *in vivo* by comparing miRNA expression in pigs infected with a virulent strain to those infected with an attenuated strain (30). These authors identified 12 miRNAs that were differentially expressed. In addition, a further study looked *in vivo* at the potential for ASFV to encode its own miRNAs and concluded that ASFV does not express miRNAs *in vivo* (31). In our study, we investigated the effect of ASFV infection on sncRNA in primary porcine alveolar macrophages *in vitro*. We found that virulent ASFV infection of primary porcine macrophages had only a small impact on host miRNAs, with only 6 out of 178 identified porcine miRNAs differentially expressed over a 16-h time period. Interestingly, we discovered an ASFV-encoded sncRNA that, when overexpressed, led to a significant reduction in ASFV replication.

(This article was submitted to an online preprint archive [32].)

## RESULTS

### ASFV infection does not induce polyadenylation or decay of cellular miRNAs.

**FIG 1:**
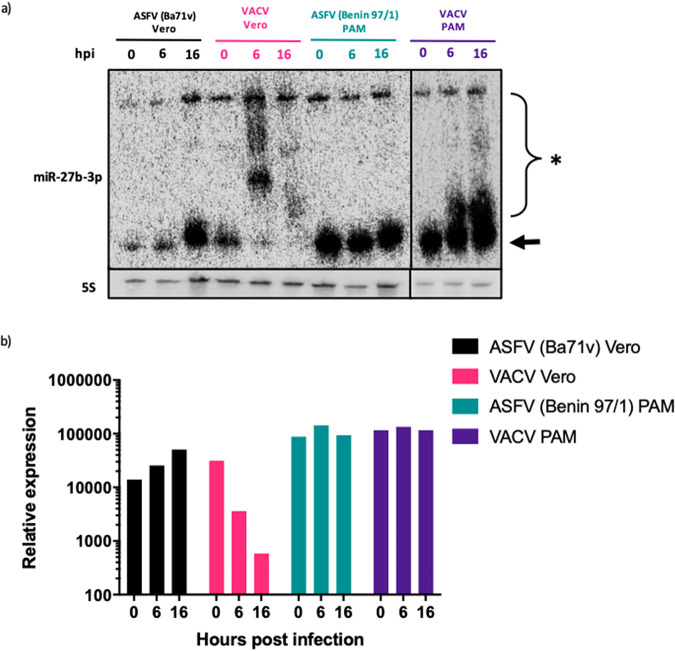
Infection with ASFV does not induce miRNA polyadenylation or decay. (a) Northern blot of RNA extracted from Vero cells and porcine alveolar macrophages (PAMs) that were infected with either ASFV or VACV. The blot was probed for miR-27b-3p and 5s and imaged on a Phosphorimager. Arrow, mature miRNA; asterisk, polyadenylated miRNA. (b) Phosphorimager quantification of miR-27b-3p expression normalized to 5S rRNA. The image and quantification is a representation of two biological repeats.

VACV, the prototypic poxvirus and NCLDV member, has been shown to induce widespread disruption of cellular miRNAs via a process of 3′ polyadenylation and decay (20, 21). To investigate if ASFV shares the ability of VACV to induce miRNA polyadenylation and decay, porcine alveolar macrophages (PAMs) were infected with the pathogenic ASFV Benin 97/1 strain and Vero cells were infected with the Vero cell-adapted ASFV strain, Ba71v. In parallel, PAMs and Vero cells were infected with VACV WR, with the samples collected and processed for Northern blotting as described. The miRNA miR-27b-3p was selected as a probe for this experiment, since it is extensively polyadenylated and degraded in VACV-infected cells (21). As expected, in VACV-infected Vero cells at 0 h postinfection (hpi), mature miR-27b-3p was seen as a lower band (Fig. 1a, arrow), but by 6 hpi this mature form was almost undetectable, and there was a higher-molecular-weight smear present in the lane, consistent with polyadenylation of the miRNA (Fig. 1a, asterisk). The higher-molecular-weight band above the smear in all lanes represents the precursor miRNA. By 16 hpi there was nearly complete decay of all forms of miR-27b-3p. In comparison, mature miR-27b-3p was present at all time points in ASFV-infected Vero cells, with no evidence of polyadenylation or decay. In VACV-infected PAMs, there was visible polyadenylation of miR-27b-3p but an absence of decay. This is likely explained by VACV being unable to undergo a complete replication cycle in PAMs (data not shown). There was no modification or reduction detected in the amount of miR-27b-3p in ASFV-infected PAMs. Levels of miR-27b-3p expression were quantified and normalized to 5S rRNA levels (Fig. 1b). This highlighted the approximately 100-fold reduction in miR-27b-3p expression in VACV-infected Vero cells, whereas in ASFV-infected Vero cells and PAMs, no reduction was detected.

**FIG 2:**
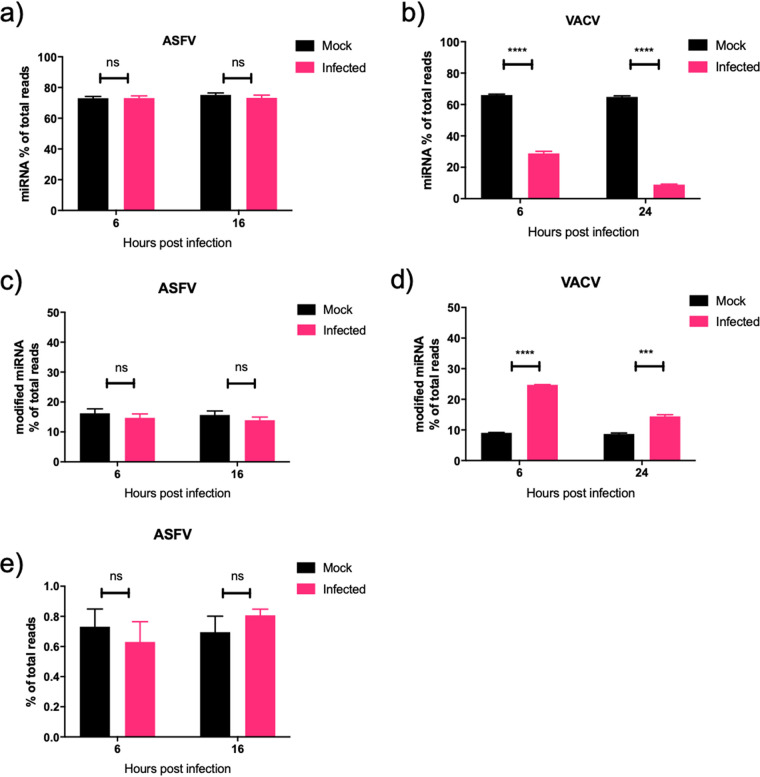
ASFV does not induce widespread polyadenylation or decay of cellular miRNAs. (a and b) The proportion of sequencing reads that mapped to known host miRNAs that were PAM infected or mock infected with ASFV (a) and in HeLa cells infected or mock infected with VACV at early and late time points (b). (c and d) The proportion of 3′ modified miRNA that contained at least 1 nontemplated nucleotide after position 19 as a proportion of total sequencing reads, in ASFV mock-infected and infected PAMs (c) and VACV mock-infected and infected HeLa cells (d). (e) Proportion of miRNA containing 3 or more 3′ nontemplated adenosine residues after nucleotide position 19 in ASFV mock-infected and infected PAMs. Data represent the means from three biological repeats, and error bars represent standard errors of the means (SEM). Data were statistically analyzed using Student’s *t* test. ns, *P* > 0.05; ***, *P* < 0.001; ****, *P* < 0.0001. Data from panels b and d were taken from supporting information from reference 18 and represent means from three biological replicates.

To look comprehensively at the effect of ASFV infection on sncRNA, we utilized small RNA sequencing. RNA was extracted and sequenced from three biological repeats of either mock- or ASFV-infected PAMs at 0, 6, and 16 hpi. Sequences aligning to 247 different mature Sus scrofa (obtained from miRbase [33]) miRNAs were obtained. Raw data were subsequently filtered to remove miRNAs with an average of fewer than 5 reads per sample; this reduced the total number of miRNAs identified to 178. These accounted for, on average, 73% of total trimmed small RNA reads. Data from a previous study investigating the effect of VACV infection on host miRNAs was used as a comparison (21). In ASFV-infected cells, there was no variation in the miRNA proportion of the total small RNA reads at both early and late time points compared to uninfected cells (Fig. 2a). This was in contrast to VACV-infected cells, which had a 30% reduction in miRNA reads at an early time point (6 hpi) and over 50% reduction at late times (24 hpi) (Fig. 2b) (21). To assess the extent of ASFV-induced 3′ modification of miRNAs, all trimmed sequences were analyzed for nontemplated nucleotide 3′ additions after nucleotide position 19. No difference was detected between mock- and ASFV-infected samples at both 6 and 16 hpi, with the proportion of reads containing miRNAs with 3′ mismatches remaining at approximately 17% (Fig. 2c). VACV infection led to a significant increase in the proportion of 3′ modified miRNA reads, which increased from 10% in mock to 25% in infected samples at 6 hpi (Fig. 2d) (21). At 24 hpi, the difference was less substantial and increased from 10% in mock to 15% in infected samples, although it was still statistically significant (Fig. 2d). In a final analysis of 3′ miRNA modification during ASFV infection, the extent of 3′ polyadenylation was examined by calculation of the proportion of miRNA reads that contained 3 or more nontemplated 3′ adenosine residues beyond nucleotide position 19. No difference was detected between mock and ASFV infected samples at either 6 or 16 hpi (Fig. 2e). The results from Northern blotting and small RNA sequencing revealed that ASFV does not share the ability of poxviruses to induce cellular miRNA polyadenylation and decay.

### ASFV infection induces rapid changes in abundance of a select number of miRNAs.

**FIG 3:**
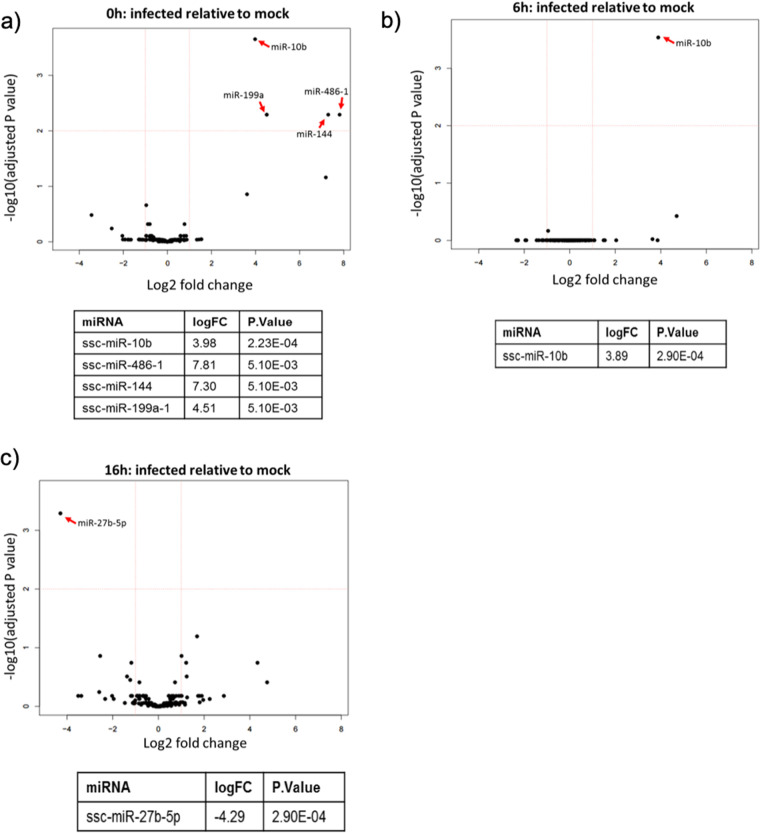
ASFV infection induces rapid changes in the abundance of a select number of miRNAs in PAMs. Volcano plots showing the differential expression of host miRNAs in ASFV-infected cells relative to mock-infected cells at 0 hpi (a), 6 hpi (b), and 16 hpi (c). The miRNAs that were differentially expressed with a significant adjusted *P* value (≤0.05) are detailed in the table below each volcano plot.

To determine if ASFV infection induces differential abundance of specific miRNAs, we used the small RNA sequencing data to analyze changes in the expression of individual miRNAs during infection relative to mock-infected cells. The filtered data (miRNA, ≥5 reads; 178 miRNAs in total) were used for this analysis. miRNAs were analyzed for differential expression at 0, 6, and 16 hpi, relative to mock-treated samples, and displayed on volcano plots (Fig. 3a to c) for each time point. The majority of cellular miRNAs were not differentially expressed in response to ASFV infection. At both 6 and 16 hpi, only one miRNA was significantly differentially expressed (Fig. 3b and c). Expression of miR-10b increased 3.89 log_2_ fold at 6 hpi, and miR-27b-5p expression decreased 4.29 log_2_ fold at 16 hpi. Interestingly, the time point with the most changes in miRNA expression was 0 h (Fig. 3a). The 0-h samples were collected after the virus had been incubated on the cells for 1 h at 37°C. Four miRNAs were significantly upregulated at this early time point: miR-10b, miR-486-1, miR-144, and miR-199a. Overall, the sequencing data indicated that ASFV infection does not have a widespread impact on host miRNA expression but does lead to rapid changes in the abundance of a small number of miRNAs.

**FIG 4:**
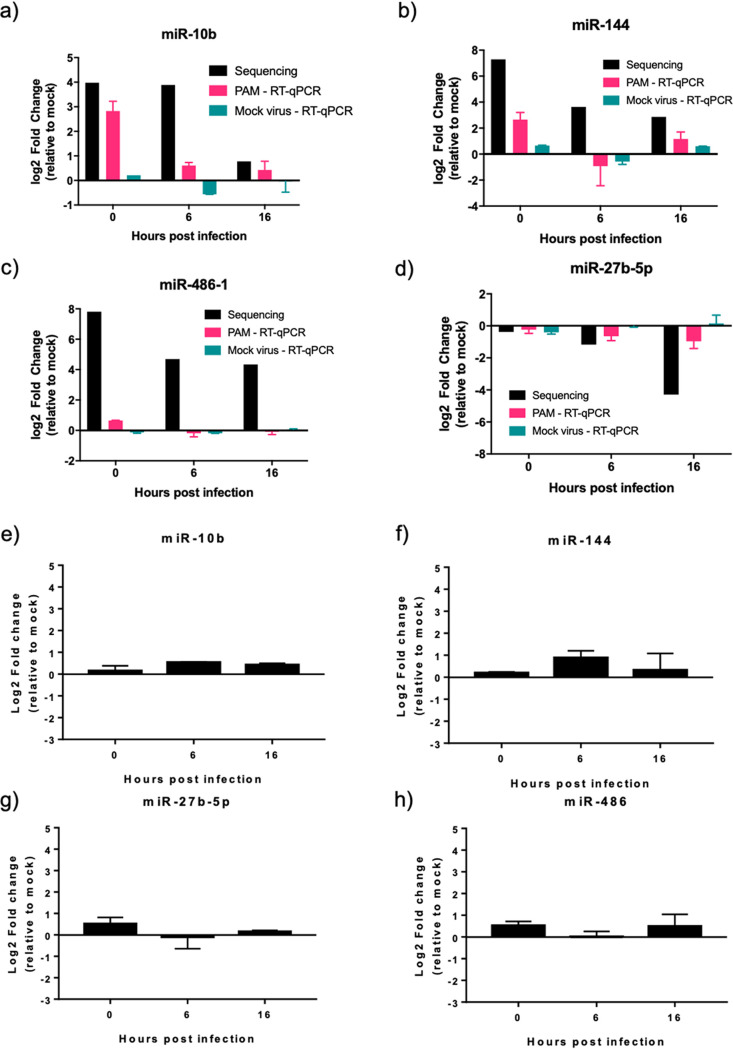
Differential expression of miR-10b and miR-144 in ASFV Benin 97/1-infected PAMs but not ASFV Ba71v-infected Vero cells. (a to d) Changes in expression of miR-10b (a), miR-144 (b), miR-27b-5p (c), or miR-486-1 (d) in PAMs infected with ASFV Benin 97/1 or after the addition of a mock virus preparation at 0, 6, and 16 hpi were measured by RT-qPCR. Fold changes from miRNA sequencing are provided for comparison. (e to h) Changes in expression of miR-10b (e), miR-144 (f), miR-27b-5p (g), or miR-486-1 (h) in Vero cells infected with ASFV Ba71v at 0, 6, and 16 hpi were measured by RT-qPCR. miRNA expression was normalized to U6 small RNA, and fold change was calculated by the *Pfaffl* method. Data represent means from 3 biological repeats, and error bars indicate SEMs. Black bars, sequencing results; pink bars, Benin 97/1-infected PAM RT-qPCR results; teal bars, mock virus-treated PAM RT-qPCR results.

To validate the miRNA expression changes identified from the sequencing analysis, quantitative reverse transcription-PCR (RT-qPCR) was used to measure the abundance of individual miRNAs. Three more biological repeats of ASFV infections were repeated in PAMs (taken from 3 different pigs), RNA was extracted at 0, 6, and 16 hpi, and miRNA expression was examined by RT-qPCR. To confirm that the miRNA expression changes were not the result of unspecific stimulation of macrophages due to cell debris in the virus preparation, we also tested miRNA expression, by RT-qPCR, after the addition of a mock virus preparation (defined as a lysate of primary porcine bone marrow cells prepared exactly as an ASFV stock would be but without any virus added). In infected PAMs, RT-qPCR validated the upregulation of miR-10b at 0 h to comparable levels detected in the sequencing (Fig. 4a); however, the upregulation at 6 hpi was not detected. Validation of miR-144 expression followed a pattern similar to that of sequencing, although the upregulation was not as substantial (Fig. 4b), with a log_2_ fold change of only 2.2 compared to 7.81 found by sequencing. RT-qPCR validation of miR-27b-5p expression showed a trend for downregulation, but, again, this was not as substantial as that detected by sequencing, with a log_2_ fold change of only −1, compared to −4 for the sequencing data at 16 hpi (Fig. 4c). RT-qPCR was unable to validate miR-486-1 upregulation (Fig. 4d). We were also unable to validate expression of miR-199a-1 due to its low expression in PAMs. With an average of only 17 miR-199a-1 reads per sample, a standard curve could not be generated. The mock virus preparation did not lead to the dysregulation of any of the tested miRNAs (Fig. 4a to d).

We also analyzed miRNA abundance in response to ASFV infection in Vero cells using the Vero cell-adapted strain, Ba71v. The expression pattern of the tested miRNAs did not match that of Benin 97/1-infected PAMs, with no substantial change in the expression of any of the studied miRNAs (Fig. 4e and f), with the log_2_ fold change not changing beyond ±1, indicating that the changes detected in PAMs with Benin 97/1 were cell type specific or strain specific. Overall, we conclude that ASFV infection has a focused impact on the abundance of cellular miRNAs. This impact is limited to the upregulation of a small number of miRNAs (miR-10b and miR-144) at very early time points during infection, specifically of Benin 97/1-infected PAMs.

### Identification of three ASFV small noncoding RNAs.

**FIG 5:**
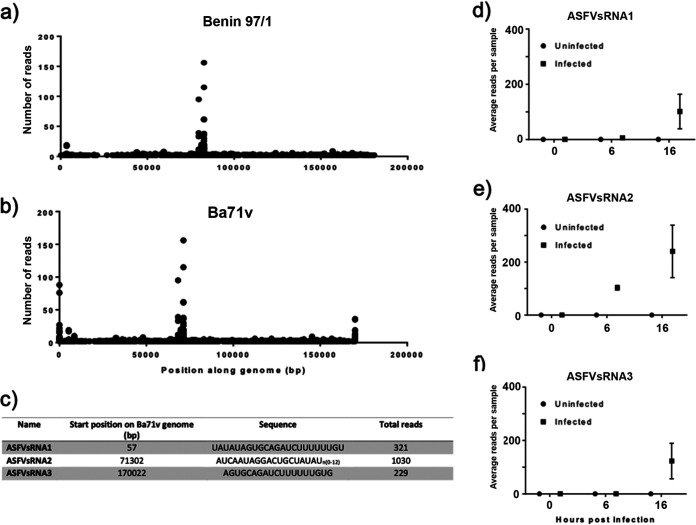
Mapping of unaligned small RNA reads to ASFV genome reveals peaks of small RNA sequences. (a) Plot showing small RNA sequencing reads mapping to the ASFV Benin 97/1 genome. The position along the genome, in base pairs, is along the *x* axis, and the number of reads is on the *y* axis. (b) Plot of reads mapping to the Ba71v ASFV genome (this sequence includes the inverted terminal repeats). (c) Table detailing the location and sequence of the peaks in reads. The average (mean) number of reads per sample at each time point is shown for ASFVsRNA1 (d), ASFVsRNA2 (e), and ASFVsRNA3 (f). Error bars indicate SEM.

As ASFV does not mirror poxviruses by disrupting the host miRNA system on a widespread scale, there remains the potential for ASFV to utilize this system to encode its own miRNAs. To investigate this possibility, we aligned small RNA reads that did not map to known Sus scrofa sequences to the ASFV Benin 97/1 genome and the Ba71v genome, as this includes fully sequenced genome termini that contain the difficult-to-sequence terminal inverted repeats (TIRs). Plotting these aligned reads along the ASFV Benin 97/1 genome (Fig. 5a) revealed a single peak of small RNA reads beginning at position 82625. This was also detected when aligned to Ba71v, beginning at base pair (bp) 71302 (Fig. 5b). In addition, the Ba71v alignment revealed two peaks of small RNA reads at bp 57 and bp 170022, indicating that these are located in the genome TIRs. We termed these 3 small RNA sequences ASFV small RNA 1, 2, and 3 (ASFVsRNA1, ASFVsRNA2, and ASFVsRNA3). Both ASFVsRNA1 and ASFvsRNA3 were only detectable at 16 hpi, with an average (mean) of approximately 100 reads per sample. ASFVsRNA2 was detectable at both 6 and 16 hpi, with an average (mean) of 240 reads per sample. The sequences of the three small RNAs are shown in Fig. 5c. Analysis of sequencing data from individual samples revealed that these small RNAs were only detected in infected samples (Fig. 5d, e, and f), supporting the hypothesis that the reads were virus derived. Due to the higher mean abundance of ASFVsRNA2 and its appearance at 6 as well as 16 hpi, this RNA was taken forward for further analysis.

### Characterization of ASFV-encoded small RNA2.

**FIG 6:**
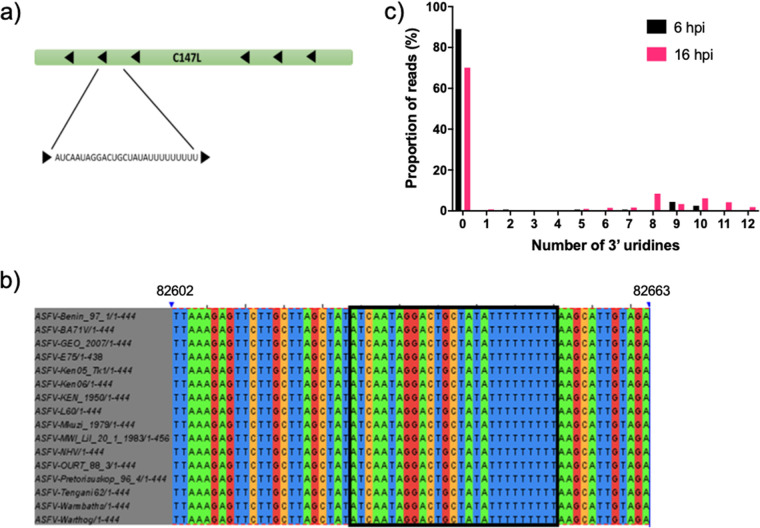
Alignment of ASFVsRNA2 to the ASFV genome. (a) Location of the ASFVsRNA2 sequence in the C147L coding region. Arrows represent coding direction. (b) Alignment of this region in multiple strains of ASFV. The boxed region highlights the ASFVsRNA2 sequence (alignment performed using Clustal Omega). Numbering is the genome position in ASFV Benin 97/1. (c) The proportion of reads and the number of 3′ uridine residues of ASFVsRNA2.

The sequence of ASFVsRNA2 aligns antisense (on the noncoding strand) to C147L (Fig. 6a), the RNA polymerase subunit 6. This region of the gene is 100% conserved in all sequenced ASFV genomes (Fig. 6b). Interestingly, ASFVsRNA2 had a variable number of 3′ U residues. A large proportion of reads did not contain any 3′ uridines, 90% at 6 hpi, which decreased to 70% by 16 hpi (Fig. 6c). The number of reads containing 1 to 12 3′ U residues increased over time from 10% at 6 hpi to 30% by 16 hpi (Fig. 6c). The first 9 U residues are templated in the viral genome (depicted in Fig. 6b), although around 10% of all reads have 10 to 12 residues at 16 hpi.

**FIG 7:**
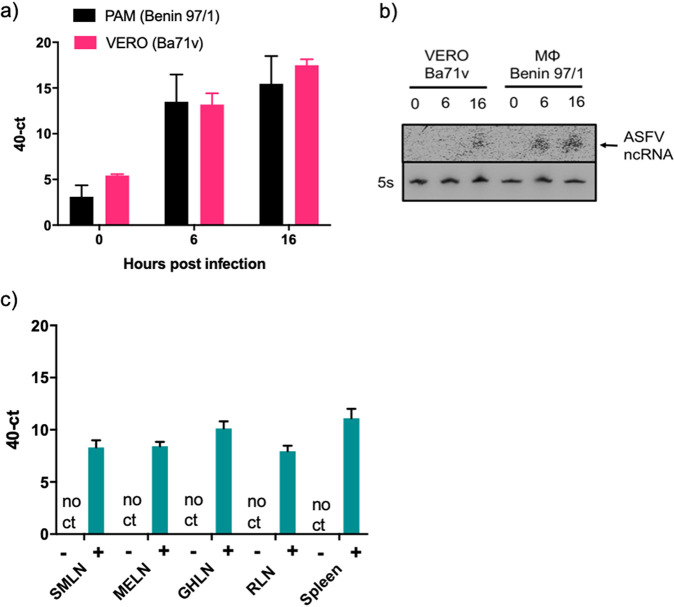
ASFVsRNA2 is expressed *in vivo*. Expression of ASFVsRNA2 was determined in ASFV-infected porcine macrophages and Vero cells at 0, 6, and 16 hpi by RT-qPCR (a) or Northern blotting (b). 5S rRNA served as a loading control. (c) RT-qPCR analysis of ASFVsRNA2 expression in tissue samples from pigs infected with ASFV OUR88/1. SMLN, submandibular lymph node; MELN, mesenteric lymph node; GHLN, gastrohepatic lymph node; RLN, renal lymph node. In panels a and c, data represent means from 3 biological replicates and error bars indicate SEMs.

The expression of the ASFVsRNA2 was validated using RT-qPCR. RNA from both ASFV Benin 97/1-infected PAMs and Ba71v-infected Vero cells was extracted at 0, 6, and 16 hpi. RT-qPCR was performed using a primer specific for ASFVsRNA2 [without the poly(U) sequence]. ASFVsRNA2 was detectable at very low levels at 0 hpi (Fig. 7a), with 40-Ct values 5 or below in both cell types. By 6 hpi, ASFVsRNA2 was readily detected, with a mean 40-Ct value of at least 12 in both cell types, and was even more abundant by 16 hpi, with the 40-Ct value increasing by at least 2. Northern blotting was also used to detect ASFVsRNA2. A radiolabeled DNA probe perfectly complementary to ASFVsRNA2 was used to perform Northern blotting on the same cellular RNA samples. The 5S rRNA was used as a loading control. The probe detected a small RNA species in infected PAMs at 6 hpi, increasing in intensity by 16 hpi. An RNA molecule of similar mobility was detected in infected Vero samples at 16 hpi (Fig. 7b).

To assess ASFVsRNA2 expression *in vivo*, RNA was analyzed from tissues taken from pigs experimentally infected with ASFV. Outbred pigs were challenged with ASFV OURT88/1 and euthanized 5 days postchallenge due to exhibiting moderate clinical signs consistent with ASF. At postmortem examination, the submandibular, mesenteric, gastrohepatic, and renal lymph nodes, as well as the spleen, were taken from three pigs. Samples of the same tissues were also taken from one uninfected animal. RNA was extracted and RT-qPCR performed using the ASFVsRNA2-specific primer. ASFVsRNA2 was not detected in any of the samples taken from the uninfected animal. ASFVsRNA2 was detectable in all samples from animals infected with ASFV, with 40-Ct values ranging from 8 to 12 (Fig. 7c), indicating that ASFVsRNA2 is produced during *in vivo* infection.

### ASFVsRNA2 does not function as an miRNA.

**FIG 8:**
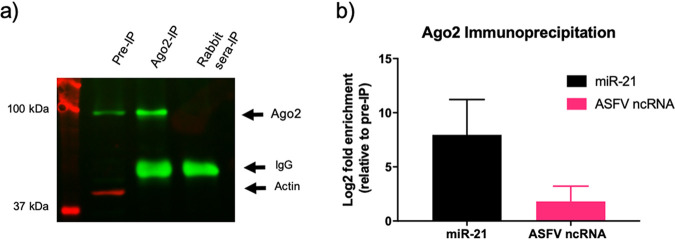
ASFVsRNA2 is not produced through an Ago2-dependent pathway. (a) Western blot of ASFV-infected PAM lysates before and after Ago2 immunoprecipitation using anti-Ago2 and anti-actin antibodies. Pre-IP, lysate before immunoprecipitation; ago2-IP, Ago2 immunoprecipitation lysate. Rabbit-IP, preimmune rabbit serum control immunoprecipitation. (b) The log_2_ fold enrichment after RT-qPCR for miR-21 and ASFVsRNA2 in the Ago2 immunoprecipitation. Expression was normalized to U6 small RNA and fold change calculated by the *Pfaffl* method. Data represent means from 3 technical replicates, and error bars indicate SEM.

To investigate whether ASFVsRNA2 is an miRNA, we investigated whether it is loaded into Argonaute 2 (Ago2), an essential protein involved in miRNA function. Lysates were harvested from ASFV-infected PAMs and incubated with either an anti-Ago2 antibody or nonimmune rabbit serum as a negative control. Antibody-protein-RNA complexes were immunoprecipitated (IP), and Western blotting was performed to identify enrichment of Ago2 in the anti-Ago2 antibody complexes compared to the nonimmune antibody complexes and the nonprecipitated cell lysate (Fig. 8a). RNA then was extracted and RT-qPCR performed for ASFVsRNA2 and the miRNA miR-21, a highly expressed miRNA known to be loaded in RISC. Data were normalized to the small U6 RNA, which is not loaded in RISC. An 8-log_2_ fold enrichment of miR-21 was detected in the complexes precipitated with the anti-AGO2 antibody, confirming the success of the IP (Fig. 8b). However, ASFVsRNA2 was very poorly enriched (log_2_ fold change of 1) after IP, indicating that ASFVsRNA2 does not function as an miRNA. We additionally used multiple RNA folding prediction programs, including miRNAFold (34), RNAfold (35), and RNAstructure (36), to predict if ASFVsRNA2 can form an miRNA stem-loop precursor. However, none of the *in silico* hairpin structures produced were convincing as miRNA hairpin precursors (data not shown), supporting the immunoprecipitation data, suggesting that ASFVsRNA2 does not function as an miRNA.

### Transfection of a mimic of ASFVsRNA2 reduces viral replication.

**FIG 9:**
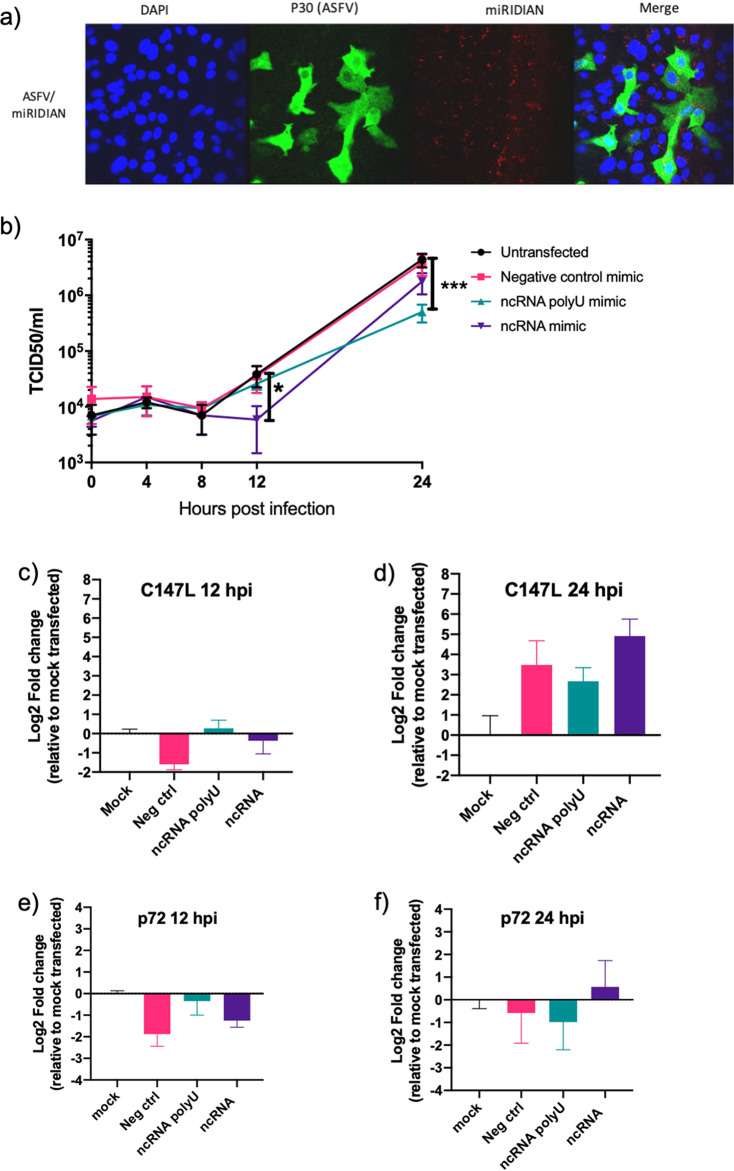
Expression of a mimic of ASFVsRNA2 leads to a reduction in ASFV replication. (a) Vero cells transfected with miRIDIAN mimic transfection control with Dy547 for 24 h were subsequently infected with ASFV Ba71v (MOI, 5). After 24 h, cells were fixed and labeled to confirm cells can be successfully transfected and infected. (b) Vero cells were transfected with RNA mimics of ASFVsRNA2, with and without poly(U) sequence. After 24 h, cells were infected with ASFV Ba71v (MOI, 5), and supernatants were taken at 0, 4, 8, 12, and 24 hpi and titrated on Vero cells. TCID_50_ was calculated by the Spearman-Karber method. Error bars indicate SEM. *, *P* = 0.0111; ***, *P* = 0.001; determined by repeated-measures two-way ANOVA. Results are representative of two biological repeats. (c to f) RT-qPCR analysis of C147L expression during the growth curve at 12 hpi (c) and 24 hpi (d) and ASFV p72 expression at 12 hpi (e) and 24 hpi (f). Expression is shown relative to mock transfection (set to 0). Data represent means from 3 technical replicates. Error bars indicate SEM.

We next sought to investigate whether ASFVsRNA2 performs a function during ASFV replication. This was achieved by synthesizing single-stranded RNA mimics of the ASFVsRNA2 with and without the 3′ poly(U) sequence. These RNA mimics were stabilized with 2′-fluoro modifications, as other sncRNAs have been shown to be functional and have targeting activity with this modification (37). The experiments were performed in Vero cells using Ba71v rather than PAMs due to the higher transfection efficiency of Vero cells. The ability of Vero cells to be both transfected with an RNA mimic and infected with ASFV was first examined. Cells were transfected with a Dy547-labeled miRNA mimic, known as miRDIAN, and at 12 h later infected with ASFV Ba71v. (After a further 24 h, cells were fixed, permeabilized, and labeled with an antibody targeted to ASFV early CP204L/p30 protein.) Analysis by confocal microscopy showed that all ASFV-infected cells (p30 positive) were also transfected with the miRNA mimic, visible as red dots on the images (Fig. 9a), indicating transfection and infection of the same cells had occurred.

A single-step ASFV growth curve was then carried out on Vero cells transfected with either the ASFVsRNA2 mimic, ASFVsRNA2 poly(U) mimic, or a negative-control mimic or were left untransfected. After 12 h, cells were infected with ASFV Ba71v, and the amount of virus present was determined at the time points shown by calculating the 50% tissue culture infective dose (TCID_50_) (Fig. 9b). The negative-control mimic had no effect on viral replication, with no difference in viral TCID_50_ at any time point compared to the nontransfected cells. At 12 hpi, ASFV replication in cells transfected with the ASFVsRNA2 mimic had an approximately 0.5-log reduction compared to the nontransfected cells. Replication in these cells recovered by 24 hpi to a level similar to that of the negative-control and nontransfected cells. Interestingly, the ASFVsRNA2 poly(U) mimic had a significant impact on replication only at 24 hpi, with an approximately 1-log reduction. Therefore, the results suggest that ASFV utilizes both the 3′ uridylated and nonuridylated forms of this virus-encoded small RNA to regulate its own replication, and that the two forms are involved at different time points during replication.

As ASFVsRNA2 lies antisense to the RNA polymerase subunit 6 gene, C147L, it is possible that it functions by targeting the C147L mRNA transcript (self-targeting). To investigate this, RNA was extracted from samples collected in parallel to the growth curve experiment (Fig. 9b) at 12 and 24 hpi. RT-qPCR analysis was performed for C147L and another ASFV gene, p72. Expression was normalized to 18s rRNA and fold change was calculated relative to infected cells that had been mock transfected. At 12 hpi, C147L mRNA abundance was not affected by the transfection of either the ncRNA mimic or the ncRNA poly(U) mimic (Fig. 9c). Transfection of the negative-control (nontargeting) mimic had a modest, approximately 1.5-log_2_ fold changed downregulation of C147L expression compared to the mock-transfected cells. At 24 hpi, transfection of all mimics, including the nontargeting control, led to an upregulation of between a 3- and 4-log_2_ fold change of C147L compared to the mock-transfected cells (Fig. 9d). In summary, no change to the C147L mRNA levels was induced by either the ncRNA mimic or the ncRNA poly(U) mimic at 12 or 24 hpi. To assess if the ncRNAs affected the expression of other ASFV genes, expression of the ASFV p72 gene was also analyzed. At 12 hpi, expression of p72 gave a pattern similar to that of C147L, with relatively little impact of ASFVsRNA2 mimic transfection, and the negative-control mimic having the largest effect, causing a 2-log_2_ fold change in downregulation relative to mock-transfected cells (Fig. 9e). At 24 hpi, there was very little difference in p72 mRNA abundance in response to transfection with any of the three mimics (Fig. 9f). Overall, this experiment indicated that transfection of ASFVsRNA2 mimics does not lead to a specific downregulation of C147L mRNA during ASFV replication or a global reduction in ASFV transcription.

## DISCUSSION

With no effective vaccine or treatment, methods to control ASFV outbreaks are limited. After reaching the European Union (EU) in 2014, ASFV has subsequently spread throughout Eastern Europe. The virus emerged in China in 2018 and has since spread rapidly across Southeast Asia, with outbreaks declared in countries including Vietnam, Cambodia, Mongolia, the Philippines, and Indonesia (38). ASFV in Southeast Asia, particularly China, which is the world’s largest pig producer, has substantially reduced food security in the region (39). Therefore, improving current ASFV vaccines and developing novel vaccines is a priority.

We sought to investigate the interaction between ASFV and sncRNAs to gain more knowledge of ASFV host-pathogen interactions. Many viruses have been found to exploit and manipulate sncRNAs. This can range from subtle effects on specific host miRNAs to widespread disruption of miRNAs, such as in the case of poxviruses (20, 21). The mechanism of VACV-mediated miRNA polyadenylation is assumed to be mediated by the virus-encoded poly(A) polymerase (PAP), VP55 (20). The viral PAP is conserved throughout the NCLDV superfamily, with the C475L gene identified as the putative ASFV PAP (40). Therefore, we first investigated whether ASFV shares the ability of poxviruses to induce miRNA polyadenylation and their subsequent decay. Both Northern blotting and small RNA sequencing revealed no evidence of widespread miRNA polyadenylation and decay in ASFV-infected cells. Therefore, this characteristic is not conserved throughout the NCLDV superfamily and may be unique to poxviruses.

In contrast to poxviruses, this study found ASFV infection has only a modest impact on host miRNAs, with only 6 miRNAs identified by sequencing to be differentially expressed during ASFV infection, and only 3 of these robustly validated by RT-qPCR. This more subtle effect on miRNAs is more common in virus infections than the widespread disruption that poxviruses induce (41). For example, pseudorabies virus-infected porcine dendritic cells led to the differential expression of only 8 miRNAs (42). A previous study examined miRNA expression in spleen and lymph node collected from pigs inoculated with a virulent and attenuated ASFV strain (19). This study identified differential regulation of 22 miRNAs in the spleen and 33 in the lymph node 3 days postinoculation. The only miRNA identified common to this study was miR-10b, which was found to be lightly downregulated between 3 and 7 dpi for animals infected with a virulent ASFV strain. It is difficult to directly compare results, as the previous study compared miRNA expression in pigs infected with a virulent strain to those infected with an attenuated strain, not uninfected animals. Additionally, our *in vitro* study focused on a single viral replication cycle over 16 h, whereas the *in vivo* experiment analyzed viral infection over multiple days. However, the identification of miR-10b as differentially expressed *in vivo* supports our theory that miR-10b plays a role during ASFV infection.

The upregulation of miR-10b occurred at 0 hpi, after the virus has been incubated on cells for only 1 h, but then rapidly decreased in abundance by 6 hpi. Further experiments using the porcine pestivirus classical swine fever virus (CSFV) also led to a similar pattern in miR-10b expression, with rapid upregulation during the first hour of infection and subsequent decrease (data not shown). Both ASFV and CSFV are enveloped viruses and have been found to enter porcine macrophages via endocytosis (43, 44). In macrophages, miR-10b is known to target ATP binding cassette transporter A1 ABCA1 (45), which is involved in the regulation of cholesterol efflux. During infection with ASFV, cholesterol remodeling is essential in the establishment of productive viral infection, and disruption of cellular cholesterol efflux leads to the impairment of virus entry and viral particles remained trapped in endosomes (46). This suggests a link between miR-10b, ABCA1, and cholesterol efflux during endocyte-mediated entry of ASFV in macrophages.

Infection of Vero cells with Ba71v did not induce the same response as infection of PAMs with Benin 97/1, indicating that the changes were either cell type specific or strain specific. This could be linked to differences in the IFN response. Vero cells are defective at producing type I IFN (47), which is the major antiviral response in mammals and, therefore, could explain the variation in miRNA response between the cell types. The difference between Vero cells and PAMs may also be a result of the different viral strains used. Compared to Benin 97/1, the Vero cell-adapted Ba71v has an 8,250-bp deletion in its genome that carries 6 copies of the MGF 360 genes and one copy of MGF-505; there is also a 3-kbp deletion at the right end of the genome (48).

A key finding of this study was identification and characterization of an ASFV sncRNA, ASFVsRNA2. Due to their large genome size, DNA viruses have the coding capacity to encode miRNAs, and many do, predominantly the nuclear replicating herpesviruses. As canonical miRNA biogenesis begins in the nucleus with host RNA Pol II transcription from the viral genome, it is assumed viral miRNA biogenesis requires a nuclear phase of viral replication. Indeed, virus-encoded miRNAs have been identified in NCLDVs that also have a nuclear phase of replication. These are in two iridoviruses: Singapore grouper iridovirus (SGIV) (49) and Tiger Frog Virus (TGV) (50). Several studies have identified the presence of ASFV genomes in the nucleus at early time points in infection (51); this nuclear phase remains poorly understood, but it indicates the possibility that ASFV encodes miRNAs.

Our study identified ASFVsRNA2 in both PAMs and tissues from pigs with ASFV (Fig. 7a to c). A previous *in vivo* study concluded that ASFV does not express viral miRNAs in experimentally infected pigs (31). However, the previous study restricted its analysis to miRNA only based on predictions of precursor miRNA structures and did not consider other classes of sncRNA. Since a number of virus families encode different classes of sncRNAs (reviewed in reference 17), we chose to sequence the small RNA fraction from ASFV-infected cells without bias, which allowed us to report the first identification of three ASFV-encoded sncRNAs. Due to a higher mean abundance and earlier detection (6 hpi), only one of these sRNAs, ASFVsRNA2, was taken forward for further characterization. ASFVsRNA2 is conserved in all sequenced isolates (Fig. 6b). However, as ASFVsRNA1 and -3 are located in the genome termini, it is unknown if these are conserved in other ASFV isolates, as the genotype I isolates Ba71v and 47/Ss/2008 remain the only isolates to have fully sequenced genome termini due to the difficulty in sequencing the TIRs. This supported our decision to focus on ASFVsRNA2.

Our research has indicated that ASFVsRNA2 does not fit the classic miRNA biogenesis pathway, as it does not have an identifiable hairpin precursor and fails to enrich in an Ago2 immunoprecipitate. Ago2 immunoprecipitation is a difficult technique to carry out, and it remains possible that our finding represents a false negative. However, it is known that a number of viral miRNAs are produced through noncanonical pathways. (52), for example, miRNAs encoded by murine gammaherpesvirus 68 (MHV68), in which the precursor miRNAs are located in a tRNA-like structure (53). Despite this variety of biogenesis pathways, miRNAs are required to be loaded into an Ago protein in order to function. Pigs, like other mammals, have been found to encode 4 Ago proteins (Ago1-4) (54), although only Ago2 is catalytically active and has the ability to cleave target mRNA (55). It has been reported that miRNAs are not sorted into distinct human Ago proteins (56), so miRNAs would be expected to be found in all Ago proteins. Therefore, our inability to detect ASFVsRNA2 enrichment in an Ago2 immunoprecipitation indicates that ASFVsRNA2 does not behave like a classical miRNA molecule.

The presence of polyuridine [poly(U)] residues on the 3′ end of ASFVsRNA2 is intriguing. The first 9 of these uridine residues are templated in the ASFV genome, although a small percentage of reads at 16 hpi have 10 to 12 uridine residues. Some of the U residues detected may, therefore, be templated, and some could arise from template slippage. It is likely that the variation in the number of templated uridine residues is a result of ASFVsRNA2 biogenesis. ASFV transcription termination takes place at a conserved motif of seven or more consecutive thymidylate residues (57), and this poly(T) motif is retained in the mRNA (58), so it is possible this poly(U) motif is the termination signal for ASFVsRNA2 transcription. Recent detailed viral transcriptome analysis of ASFV has indicated that this motif is the termination sequence for the gene upstream of C475L, C135R (59). Therefore, it is possible ASFVsRNA2 is generated through modification of the C135R mRNA transcript or as a result of readthrough transcription of this transcript. In VACV, readthrough transcription is common due to the imprecise control of transcription termination of intermediate and late genes (60). ASFV exerts stricter control over transcription termination than VACV, with termination of all gene classes occurring at a poly(T) motif, and readthrough transcription does not appear to be a common feature. In support of this, scrutiny of transcriptome sequencing (RNA-seq) reads from ASFV replication transcriptome analysis revealed little evidence of readthrough transcription (61). Readthrough transcription has been described from the Y118L gene (62), and no RNA-seq reads from these genes were detected in our study. Therefore, if ASFVsRNA2 was an artifact of readthrough transcription, it seems unlikely that this would be the only sequencing read detected at this abundance.

The nontemplated additions indicate a modification to ASFVsRNA2, as the main function of noncoding RNA uridylation is to promote its degradation (63). However, it can also have a number of other functions. For example, uridylation of the miRNA let-7 precursor is required for let-7 biogenesis (64). It can also control the activity of noncoding RNA, for instance, uridylation of miR-26 prevents it from binding to its target interleukin-6 transcript but does not affect miRNA stability (65). The functional significance of the uridine residues, templated or not, on the 3′ end of ASFvsRNA2 is currently unknown, although our work has indicated it is of importance.

Overexpression of ASFVsRNA2 reduced ASFV replication in Vero cells, suggesting it has a role in the control of viral replication. This relatively modest effect, between 0.5- and 1-log reduction, is consistent with sncRNA gene targeting, since miRNAs usually regulate gene transcript levels by less than 2-fold (66). Even though we do not believe ASFVsRNA2 to be an miRNA, the predominant function of all classes of sncRNAs is to induce gene silencing (67). Therefore, we would also expect ASFVsRNA2 to have only a modest effect. Many viruses are known to encode activators and repressors to finely control their life cycle in order to replicate in the face of the host immune response. An interesting example of this is the human cytomegalovirus (HCMV) miRNA miR-UL112-1, which has been found to downregulate multiple viral genes involved in replication, leading to a decrease in genomic viral DNA (68). There is no suitable porcine cell line in which to confirm the reduction in replication seen in Vero cells.

As ASFVsRNA2 has perfect complementarity to ASFV C147L, the region where it is derived, we investigated whether this small RNA was self-targeting. Self-targeting is already known to occur for virus-encoded miRNAs. For example, the EBV-encoded miRNA, miR-BART-2, is encoded antisense to its target, the viral DNA polymerase BALF5 (69). It is hypothesized that this allows EBV to regulate its transition from latent to lytic infection. ASFV does not have a latent stage of infection but does have strict temporal control of gene expression. Therefore, it is possible that ASFV uses ASFVsRNA2 to exert subtle control over C147L expression, an RNA polymerase subunit, to aid its strict control over gene expression. However, RT-qPCR analysis did not find any significant effect of ASFVsRNA2 mimic transfection on C147L expression during a growth curve. Therefore, it appears that ASFVsRNA2 does not function by disrupting the stability of the C147L transcript.

In summary, we have found that ASFV infection has a very modest impact on host sncRNA and instead utilizes this system to encode its own, ASFVsRNA2, which retards viral replication. The discovery of an ASFV-encoded sncRNA has added another level to the knowledge of ASFV replication. Furthermore, the finding that ASFV induces little disruption to the host miRNA system opens up the possibility of utilizing host miRNAs to target and downregulate ASFV genes, potentially aiding the development of the next generation of ASFV vaccines.

## MATERIALS AND METHODS

### Cells and viruses.

African green monkey epithelial cells (Vero) were grown in Dulbecco’s modified Eagle’s medium (DMEM) (Life Technologies) containing 100 U/ml penicillin, 100 μg/ml streptomycin (Sigma), and 10% fetal bovine serum (FBS) (Life Technologies). Cells were maintained at 37°C with 5% CO_2_ and were passaged regularly to maintain viability. Primary porcine cells were derived from 4-week-old Large White outbred pigs. Porcine alveolar macrophages (PAMs) were obtained by lung lavage with phosphate-buffered saline (PBS) and maintained in RPMI 1640 GlutaMAX (Life Technologies) containing 100 U/ml penicillin, 100 μg/ml streptomycin (Sigma), and 10% porcine serum (BioSera). Bone marrow cells were prepared from femur bones and maintained in Earle’s balanced salt solution (EBSS) with 100 U/ml penicillin, 100 μg/ml streptomycin (Sigma), 10% porcine serum (BioSera), and 10 mM HEPES. The pathogenic ASFV Benin 97/1, previously described in reference 48, was grown in primary porcine bone marrow cells and the tissue culture-adapted ASFV BA71V, previously described in reference 70, was grown in Vero cells. The mock virus preparation used in Fig. 4 was prepared in parallel with a pathogenic ASFV Benin 97/1 virus stock on primary porcine bone marrow cells, but no virus was added to the cells. Experiments involving VACV used the Western Reserve (VACV-WR) strain, prepared by purification on 36% (wt/vol) sucrose cushion. Experiments involving CSFV used the Brescia strain, kindly provided by Julian Seago (The Pirbright Institute).

### Animal experiments and ethics statement.

Animal experiments were carried out under the Home Office Animals (Scientific Procedures) Act (1986) (ASPA) and were approved by the Animal Welfare and Ethical Review Board (AWERB) of The Pirbright Institute. The animals were housed in accordance with the Code of Practice for the Housing and Care of Animals Bred, Supplied or Used for Scientific Purposes, and bedding and species-specific enrichment were provided throughout the study to ensure high standards of welfare. Through careful monitoring, pigs that reached the scientific or humane endpoints of the studies were euthanized by an overdose of anesthetic. All procedures were conducted by Personal License holders who were trained and competent and under the auspices of Project Licences. Female Landrace × Large White (Yorkshire) × Hampshire pigs were obtained from a high-health farm in the United Kingdom. Animals were challenged intramuscularly in the rump with 10,000 hemabsorbing units of the OUR T88/1 strain of ASFV. Tissue samples were collected from three pigs at postmortem, 5 days postchallenge.

### Preparation of RNA samples.

Confluent 6-well plates of Vero cells and PAMs were mock infected or infected with ASFV or VACV at a multiplicity of infection (MOI) of 10 for 1 h at 37°C. The inoculum was removed (0-h time point), cells were washed 3× in PBS, and medium, containing 2.5% serum, was replaced. At 0, 6, and 16 hpi, cells were harvested into an appropriate volume of QIAzol lysis reagent (Qiagen) and RNA prepared using the miRNeasy minikit (Qiagen). For RNA extraction from animal tissues, the tissues were harvested into RNAlater (Life Technologies) and stored at −80°C. Fifty milligrams of tissue was added to 700 μl Qiazol lysis reagent (Qiagen) and homogenized using tissue grinding lysate matrix beads (MP Biomedicals). RNA was prepared using the miRNeasy minikit (Qiagen), including an on-column DNase digest (Qiagen).

### Northern blot analysis.

Northern blotting was carried as described in reference 21. Briefly, 5 μg RNA was mixed with 2× Tris-borate-EDTA (TBE)-UREA buffer (Novex) and heated at 70°C for 3 min. Samples were run on a 15% polyacrylamide TBE-UREA gel (Bio-Rad). The gel was then transferred to a solution of 0.5× TBE containing 10,000× SYBR gold (Invitrogen) and visualized under UV light to check for equal loading. The RNA was transferred to a Hybond N^+^ membrane (Amersham) using a semidry transfer machine and cross-linked as described in reference 71. DNA probes perfectly complementary to miR-27b-3p, ASFVsRNA2, and 5S rRNA were prepared by labeling with ^32^P using the mirVana probe and marker kit (Ambion). Membranes were prehybridized in ULTRAhyb hybridization buffer (Ambion) for 1 h at 42°C prior to incubation with a ^32^P-labeled DNA probe overnight at 42°C. Membranes were then washed twice in 2× SSC (1× SSC is 0.15 M NaCl plus 0.015 M sodium citrate) with 0.1% SDS for 15 min and laid against a phosphorimage screen for 4 to 6 h. Labeling was detected using a Typhoon FLA 7000 phosphorimager (GE Healthcare). To strip the membrane for reprobing, it was washed in a solution of boiling 0.1% SDS for 30 min.

### Small RNA sequencing.

Integrity of the RNA was measured on a Bioanalyzer (Agilent), with all samples having a RIN value of 8 or above. Small RNA sequencing libraries were prepared using the CleanTag small RNA kit (TriLink). Libraries were pooled, gel purified on a 5% polyacrylamide TBE gel (Bio-Rad), and sequenced on an Illumina HiSeq (Edinburgh Genomics). An average of 7,013,808 ± 3,522,153 reads per sample was generated. The quality of reads was assessed using FASTQC and adapters trimmed using cutadapt software. Sequences were collapsed within each sample to generate a nonredundant set of fasta sequences. Singletons were not included. The reference used for alignment was version 10.2 of the Sus scrofa genome obtained from Ensembl; only full-length perfect match (FLPM) sequences were counted. Sequences aligning to the genome were subsequently used as input for a mirDeep2 analysis. Alignments were performed using a noncurrent version of bowtie-based Perl script (mapper.pl) that forms part of the mirDeep2 software package. The mirDeep2 version was 2.0.0.4, and the bowtie version was 0.12.5. Parameters used were -*o 20 -l 17 -r 100 -c*. The analysis used Sus scrofa mature (3p and 5p forms) and precursor sequences obtained from mirBase (release 21). Small RNA reads that did not map to Sus scrofa sequences on mirBase were aligned to the ASFV BA71V genome (GenBank accession no. U18466.2) and ASFV Benin 97/1 genome (GenBank accession no. AM712239.1). Sequencing data were deposited in NCBI GEO database under accession no. GSE115512.

### Differential expression analysis.

Initial raw counts were filtered to include only those with an average of 5 reads or more. The counts within each sample were converted to abundances, which were (i) multiplied by one million to generate a read set and (ii) one count added to all to preclude zero counts instances, and (iii) the resultant values were converted to log_2_ and quantile normalized. Pairwise comparisons of sample groups were performed on the normalized tag counts using linear modeling (Bioconductor *limma* package). A series of 6 group-wise comparisons using empirical Bayesian approaches was undertaken to identify differences (fold changes). Significance values are controlled for false discovery, yielding a more rigorous adjusted *P* value.

### RT-qPCR.

For small RNA analysis, cDNA was generated using the miScript II RT kit (Qiagen), and for mRNA analysis, cDNA was generated using the oligo(dT)_20_-primed SuperScript III first-strand synthesis system for RT-PCR (Invitrogen). qPCR was performed using the miScript SYBR green PCR kit (Qiagen) using miScript primer assays for miRNAs and using the QuantiTect SYBR green PCR kit (Qiagen) for mRNA in a Stratagene Mx3005P qPCR machine (Agilent). All qPCRs were performed in duplicate. Hs_RNU6-2_11 miScript Primer was used as a reference gene for sncRNA data normalization, and 18S rRNA was used for mRNA data normalization. The PCR efficiency of each primer was determined by standard curve, and the log_2_ fold change was calculated by the *Pfaffl* method.

### Immunoprecipitation and Western blotting.

Lysates were prepared by washing cells 2× in ice-cold PBS before addition of an appropriate volume of radioimmunoprecipitation assay lysis buffer (ThermoFisher) supplemented with protease inhibitor (Complete protease inhibitor cocktail tablets; Roche) and, for immunoprecipitation, RNase inhibitor (ThermoFisher). Protein concentrations were determined using the bicinchoninic acid protein assay kit (Thermo Scientific Pierce). For immunoprecipitation, equal amounts of protein were incubated with either a rabbit polyclonal anti-Ago2 antibody or preimmune rabbit sera overnight at 4°C. This was followed by a 1-h incubation at room temperature with 25 μl Pierce protein A magnetic beads (Thermo Scientific). The beads were washed and resuspended and then either prepared for Western blotting or added to QIAzol lysis reagent for RNA extraction. For Western blotting, lysates or bead suspensions were prepared by mixing 20 μg of protein with 2× protein sample loading buffer (Li-Cor) and heated to 98°C for 5 min. Samples were loaded onto a 15% polyacrylamide resolving gel layered with a 5% stacking gel, alongside a prestained protein ladder (Bio-Rad), and the proteins separated by electrophoresis. Proteins were transferred to a polyvinylidene difluoride membrane using a wet transfer technique. Membranes were first blocked for 1 h at room temperature in a 1:1 mixture of PBS and Odyssey blocking buffer (Li-Cor) and then incubated with the primary antibodies diluted in Odyssey blocking buffer and 0.1% Tween at 4°C overnight. Membranes were washed 4× for 5 min in PBS containing 0.1% Tween before being incubated with the secondary antibodies diluted in Odyssey blocking buffer and 0.1% Tween for 45 min at room temperature. Primary antibodies used were rabbit anti-Ago2 (kindly provided by F. Gray), rat anti-HA, rabbit anti-actin (Cell signaling), and mouse anti-actin (Cell Signaling). The secondary antibodies were DyLight 680 and 800 (Cell Signaling). Membranes underwent a further 4 5-min washes in PBS-Tween and were then visualized on a G:Box (Syngene).

### Plasmid and RNA mimic transfection.

Vero cells were seeded at an appropriate density 24 h before transfection. Both plasmid DNA and RNA mimics were transfected using Transit-X2 (Mirus) by following manufacturer’s protocol. In brief, 500 ng DNA and/or 10 μM RNA mimic was diluted in 50 μl Opti-MEM (LifeTech), and 1.5 μl transfection reagent was added and incubated for 15 min at room temperature before adding to 1 well of a 24-well plate. The single-stranded RNA mimics were synthesized (Sigma) with 5′-phosphorylation and 2’-fluoro modification for stability. The sequences of the RNA mimics were the following: ASFVsRNA2 mimic, AUCAAUAGGACUGCUAUA; ASFVsRNA2 poly(U) mimic, AUCAAUAGGACUGCUAUAUUUUUUU; negative control, UUCUCCGAACGUGUCACGU. The miRIDIAN microRNA mimic transfection control with Dy547 was sourced from Dharmacon. Mimics were transfected to give a final concentration of 25 nM per well.

### One-step growth curve.

Vero cells were transfected with RNA mimics as described above and incubated for 14 h at 37°C. Cells were then infected with ASFV Ba71v at an MOI of 5 for 1 h at 37°C and washed 3×, and then medium was replaced. Virus was harvested at 0, 4, 8, 12, and 24 hpi by scraping the cells into the medium and freeze-thawing 3×. Viral titers were determined by immunofluorescence TCID_50_ assay on Vero cells using an antibody against ASFV P30 protein. TCID_50_ was calculated using the Spearman-Karber method.

### Immunofluorescence.

Cells were fixed after 3 washes in ice-cold PBS with 10% formalin and incubated for 30 min at room temperature. Cells were washed again 3× in PBS, permeabilized by the addition of 0.2% Triton X-100 diluted in PBS for 5 min at room temperature and washed a further 3× in PBS. The primary antibody (mouse anti-ASFV P30) was diluted in PBS with 2% FBS and incubated with the cells in a humidity chamber for 1 h. Cells were then washed 3× in PBS with 2% FBS and incubated with secondary antibody (Alexa Fluor 488-conjugated goat anti-mouse IgG), and fluorescently tagged phalloidin (Molecular Probes), in a humidity chamber for 1 h. Coverslips were stained with 300 nM 4′,6-diamidino-2-phenylindole (DAPI) (Life Technologies) for 5 min and then rinsed 3× in PBS, with a final rinse in distilled H_2_O before being mounted onto a microscope slide using Vectashield (Vector Labs).

### Data availability.

Raw data are available from the NCBI GEO database under accession no. GSE115512.
